# The Role of Lipid Domains in Bacterial Cell Processes

**DOI:** 10.3390/ijms14024050

**Published:** 2013-02-18

**Authors:** Imrich Barák, Katarína Muchová

**Affiliations:** Institute of Molecular Biology, Slovak Academy of Sciences, Dúbravská cesta 21, Bratislava 84551, Slovakia; E-Mail: katarina.muchova@savba.sk

**Keywords:** lipid domains, bacterial cell division, sporulation, cardiolipin, phosphatidylglycerol

## Abstract

Membranes are vital structures for cellular life forms. As thin, hydrophobic films, they provide a physical barrier separating the aqueous cytoplasm from the outside world or from the interiors of other cellular compartments. They maintain a selective permeability for the import and export of water-soluble compounds, enabling the living cell to maintain a stable chemical environment for biological processes. Cell membranes are primarily composed of two crucial substances, lipids and proteins. Bacterial membranes can sense environmental changes or communication signals from other cells and they support different cell processes, including cell division, differentiation, protein secretion and supplementary protein functions. The original fluid mosaic model of membrane structure has been recently revised because it has become apparent that domains of different lipid composition are present in both eukaryotic and prokaryotic cell membranes. In this review, we summarize different aspects of phospholipid domain formation in bacterial membranes, mainly in Gram-negative *Escherichia coli* and Gram-positive *Bacillus subtilis*. We describe the role of these lipid domains in membrane dynamics and the localization of specific proteins and protein complexes in relation to the regulation of cellular function.

## 1. Introduction

In the past, bacterial cells were thought of as vessels without internal organization, with proteins, mRNA, chromosomes and other soluble compounds dispersed somewhat randomly inside of a membrane-enveloped cytoplasm. This view has gradually changed, thanks mainly to recent advancements in imaging technology and the development of better fluorescent proteins and dyes. It has become clear that a large amount of the cytoplasmic material is specifically localized in the cell and that this localization is crucial for the correct operation of different cellular processes. It has become apparent that membranes play an important role in establishing specific localization patterns of membrane proteins within the cell [1,2]. The earlier fluidic mosaic model [3] assumed that lipids are homogeneously distributed in the membrane. This assumption was recently called into question when it was discovered that many different proteins are localized to specific sites in the bacterial membrane and that the membrane contains domains that differ in their lipid composition. The localization of proteins to specific sites in the cell membrane may be governed by chemical factors, for example the phospholipid composition of the lipid domains [4,5] and rafts [6], by different physical characteristics of the membrane, e.g. its degree of negative or positive curvature [7,8] or its electrical potential [9], and by the different lipid composition of membranes belonging to differentiated cell compartments. Taken together, all these factors show that the formation of lipid domains in cell membranes allows cellular asymmetry to be developed; this asymmetry is crucial for the life cycle and for many different vital processes in bacteria.

## 2. Bacterial Membranes

The two main classes of bacteria, Gram-positive and Gram-negative, differ in the composition of their cell wall and membranes. The cell wall of Gram-positive bacteria consists of a thick layer of peptidoglycan and a single membrane. The organization of a Gram-negative bacterial cell wall is more complex. It is made up of two membranes, an outer membrane and an inner membrane; these two membranes sandwich a region known as the periplasmic space which has very important functions in the survival and operation of the cell. This region acts as a buffer between the very different conditions of the external environment and the interior of the bacterium. It contains specialized transport proteins and sensory proteins, which detect conditions in the bacterium’s surroundings and help to determine an appropriate response to them.

Bacterial membranes are primarily comprised of different phospholipids. Bacterial phospholipids consist of a polar, hydrophilic head and a hydrophobic tail. The hydrophilic head appears as an electron-dense region in transmission electron microscopy while the tail, which is buried within the membrane, forms an electron-transparent region. Thus a thin section of membrane, when viewed by an electron microscope, appears as two parallel, thickly stained lines separated by an almost transparent region. In the membrane of Gram-positive bacteria and in the inner membrane of Gram-negative bacteria, the phospholipids are arranged fairly evenly on either membrane leaflet, forming a symmetric lipid bilayer. The outer membrane of Gram-negative bacteria, in contrast, has an asymmetric arrangement of phospholipids: most of the phospholipids are located in the inner leaflet of the membrane while the outer leaflet contains some phospholipids, but also proteins and modified lipid molecules termed lipopolysaccharides (LPS). The transmembrane proteins found in the outer membrane predominantly have a β-barrel fold whereas the inner membrane harbors mainly α-helix-based transmembrane proteins. The outer membrane provides a physical barrier from the outside world, but it also must maintain selective permeability for the uptake of nutrients to support the cell’s physiological functions and to allow virulence factors to be released.

## 3. Formation of Lipid Domains

### 3.1. Cardiolipin Domains

The recent development of new lipid fluorescent probes and localization experiments, which showed that many proteins tend to associate with specific regions of the membrane, led to the discovery of lipid domains, that is, regions of the membrane domains that differ in their lipid composition. The general view of membranes described by the original fluid mosaic model [3] was first questioned for eukaryotic cells and there is now a lot of evidence for the existence of lipid rafts and other specific lipid domains. It became clear somewhat later that lipid domains also exist in bacterial membranes and that these domains have a crucial role in the specific localization of protein and protein complexes [1,10,11]. Although the discovery of lipid domains mostly took place in two model organisms—Gram-negative *E. coli* and Gram-positive *B. subtilis*—lipid domains are also likely to exist in all other bacterial species. The membranes of both model organisms contain the same principal phospholipids, but in very different amounts. The major glycerolphosphate-based lipids of *E. coli* include the zwitterionic phosphatidylethanolamine (PE, 70% of the membrane), the anionic phosphatidylglycerol (PG, 20%), and cardiolipin (CL, 10%). In *B. subtilis*, on the other hand, PE comprises only 20%, PG, 40%, and CL 25%; the remaining 15% is the zwitterionic lipid lysyl-phosphatidylglycerol [12]. These numbers are only approximate, however, because the phospholipid composition of the membrane can change dramatically during the bacteria’s life cycle, as Gidden *et al.* showed using matrix-assisted laser desorption/ionization time-of-flight (MALDI-TOF) mass spectrometry and TOF/TOF tandem mass spectrometry for *E. coli* and *B. subtilis* [13]. During stationary growth of *E. coli*, the mass spectra indicated an increase in the relative amount of saturated fatty acids. In *B. subtilis*, the phospholipid composition remained relatively unchanged during exponential growth, but the amount of PG slightly decreased while the amount of PE slightly increased during stationary growth. It has also been shown that the phospholipid composition of the forespore membrane changes during early stages of sporulation in *B. subtilis* [14].

**Figure 1:**
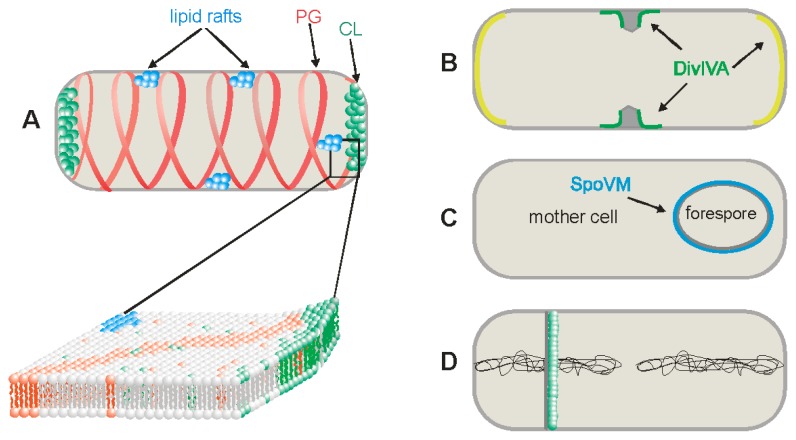
Schematic presentation of different chemical and physical cues for protein localization into *Bacillus subtilis* membranes. (**A**) Presentation of three types of lipid domains—lipid rafts, cardiolipin (CL) and phosphatidylglycerol (PG) domains; (**B**) Negative curvatures membrane recognized by DivIVA. The highest negative curvature is near the forming septum, marked in green and moderate negative curvatures are at the poles, marked in yellow; (**C**) The positive curvature membrane, recognized by SpoVM, is located at the outer surface of the forespore in sporulating *B. subtilis*, marked in blue; (**D**) Asymmetrical *de novo* lipid synthesis in the sporulation septum from the mother cell side.

The first lipid domains discovered were cardiolipin domains in the membranes of *E. coli* [4,15,16] and *B. subtilis* [17]. CL has a dimeric molecular structure in which two phosphatidyl moieties are linked by a glycerol; CL can be specifically detected in cell membranes by the hydrophobic fluorescent dye 10-*N*-nonyl acridine orange (NAO). More than two decades ago, NAO was developed for the visualization of CL rich mitochondria in eukaryotic cells [18]. It was later shown that NAO also binds to the cardiolipin of the *E. coli* and *B. subtilis* membranes and localizes to their polar and septal regions (Figure 1A) [4,16,17]. NAO has a higher affinity for CL than for other anionic phospholipids [19,20] and association with CL but not with other phospholipids induces a green to red shift in its fluorescence emission maximum [4].

It is becoming clear that the existence of CL domains in bacterial membranes at cell poles and at the site of septation influences the polar localization of many proteins, which is necessary for carrying out their specific functions. However, it is not clear how these domains form. It has been proposed that membrane curvature may be a crucial factor in the polar localization of high-intrinsic-curvature lipids like CL. It is thought that osmotic pinning of the membrane to the cell wall naturally produces microphase separation, whose aggregate sensitivity to cell curvature can support the spontaneous and stable localization of CL to both poles [21]. According to this model, there ought to be a critical concentration of CL below which domains will not form and hence polar localization will not take place. This is consistent with experiments showing that in *E. coli* cells with reduced CL concentrations, CL and the osmoregulatory protein ProP fails to localize to the cell poles [22,23]. Interestingly, significantly enhanced levels of CL have been found in the membrane of *E. coli* minicells, which are derived from the cell poles [24], and in the engulfment and forespore membranes of *B. subtilis* cells during sporulation [25].

### 3.2. Phosphatidylglycerol Domains

In experiments using *B. subtilis*, phospholipid specific dyes of the FM series, including FM4-64, FM1-43 and FM5-95, are found to be distributed on helical structures along the cell length (Figure 1A) [5], when *E. coli* cells are used, the dyes appear in bands or dots but not in discernible helical structures [5,26]. These FM dyes are cationic styryl compounds. They have a net charge of +2, which ordinarily prevents them from passing through cell membranes [27], and they are therefore expected to preferentially associate with membranes enriched in negatively charged phospholipids such as PG and CL. This implies that the fluorescence spirals observed in *B. subtilis* after treatment with these dyes represent membrane structures that are richer in these two phospholipids than the rest of the membrane. These findings raise a crucial question how such striking helical lipid structures can form? It is possible that they linked to the cytoskeletal protein structures and/or to the biosynthesis of cell wall peptidoglycan. It was shown that in protoplasted cells, devoid of the peptidoglycan layer, helix-like lipid structures are not preserved [28]. Specific lipid domains are also missing in cells depleted of MurG, an enzyme involved in peptidoglycan synthesis, indicating a link between lipid domains and peptidoglycan synthesis [28]. The helical FM structures are absent in *E. coli* cells but this is inconclusive: it is possible either that these lipid helices are not present in this organism or that the additional outer membrane layer of the Gram-negative cell obscures observation of them.

Additional information on the composition of these spirals comes from gene disruption experiments. The *B. subtilis pgsA* gene encodes phosphatidylglycerol phosphate synthase, an essential enzyme in the biosynthesis of PG and CL [29]. In cells in which the biosynthesis of PG and CL phospholipids was disrupted, the FM dyes localized to the periphery of the cells, at the cell poles and at the site of septation. Clear lipid helices were discernible only in cells in which *pgsA* expression had been induced [5]. It appears, therefore, that the formation of these lipid helices is partly or wholly dependent on the presence of PG and CL in the membrane. Because the CL-specific NAO dye is preferentially distributed in the septal regions and at the poles of *B. subtilis* cells, the principal component of the helical structures is likely to be PG (CL may still be present at much lower levels, however). Because cells lacking both PG and CL are not viable, it is not possible to verify the composition of these helices by excluding them. Cells depleted of these components by removal of the *pgsA* inducer do survive for several hours, but this is because their function can be partially substituted by their anionic precursors, phosphatidic acid and CDP-diacylglycerol, which accumulate in the absence of phosphatidylglycerol phosphate synthase [10]; these species are likely recognized by the positively charged FM dyes.

CL domains have been shown to serve as cues for the localization of proteins to specific sites in the cell, specifically the poles and septation sites. Similarly, overexpressed GFP-MinD has been shown to colocalize with PG helical structures in *B. subtilis*. The MinD protein is part of the Min system division site selection machinery, which is present in most rod shaped bacteria [30,31]. Two basic types of Min system are known. One is found in Gram-negative bacteria such as *E. coli* and a second type is present in the Gram-positive *B. subtilis*. In both systems, it is thought that a concentration gradient of Min system proteins serves as a measuring device for defining the mid-cell site of cell division. In *E. coli*, this is achieved through the oscillation of the Min proteins from pole-to-pole on a helical trajectory [32–34]. In *B. subtilis*, protein oscillation is not observed, so different mechanisms are thought to operate. Furthermore, a DivIVA/MinJ complex, which attracts MinCD to the cell poles, takes the place of *E. coli*’s MinE protein [35,36]. Most other rod-shaped bacteria have either a MinCDE or a MinCD/MinJDivIVA system, so it is generally assumed that they follow cell-division-site-recognition strategies similar to those of either *E. coli* or *B. subtilis*. The discovery of lipid helical domains recognized by MinD in *B. subtilis* allows the models for cell division site selection in the two systems to be brought closer together. A longitudinal concentration gradient of MinCD, a cell division inhibition complex, can be built up either by oscillation of these proteins on lipid helices or by preferential attraction to the poles along an anionic helical track. In both cases, this gradient is capable of defining the mid-cell plane with high precision for accurate cell division.

The molecular details of how this MinCD complex can bind to helical anionic phospholipid domains have also been resolved. MinD, an ATPase, appears to associate reversibly with the cytoplasmic membrane using its *C*-terminal residues [37,38]. The sequence at the *C*-terminus of MinD (the membrane targeting sequence or MTS) is conserved across archaea, eubacteria, and chloroplasts, and has been shown to mediate interactions between MinD and membrane lipids *in vitro* and to be essential for membrane localization *in vivo* [37]. The MTS of *E. coli* appears to bind preferentially to anionic phospholipids and several of the hydrophobic residues within this sequence insert into the cell membrane lipid bilayer [39]. The MTS of *B. subtilis* MinD is three amino acid residues longer than that of *E. coli*, suggesting that it may have a higher affinity for the membrane [37].

Although, this MTS is not fully resolved in any of the three crystal structures of MinD homologues known to date, there is still good evidence to suggest that it forms an amphipathic α-helix, at least upon membrane binding. One face of this helix is lipophilic while the other face is positively charged [37]. This amphipathic α-helix could then align itself parallel to the membrane surface so that its hydrophobic face interacts with the lipid acyl chains while the cationic side chains interact with the anionic phospholipid head groups [40]. These favorable electrostatic interactions would therefore explain the preference of MinD for membranes enriched in anionic phospholipids.

The importance of the MTS for the specific membrane localization of *B. subtilis* MinD was shown by the deletion of this sequence [5]. In this mutant, MinD failed to exhibit a helical pattern of membrane localization and its signal was diffusely distributed throughout the cytoplasm. This failure of MinD to localize to the membrane correlated with loss of MinCD’s function as a cell division inhibitor.

The PG helical domains may also serve as cues for the localization of other proteins with similar MTS domains. For example, the key cell division protein, FtsZ marks the future division site by forming a highly oligomeric structure, called Z-ring. This protein, together with its membrane-binding partner, FtsA, can form dynamic helical structures in vegetatively growing and sporulating *B. subtilis* cells [41,42]. FtsA, like MinD, has an amphipathic, positively charged α-helix that is required for membrane localization [43]. Preferential binding to helical PG domains therefore provides an obvious explanation for the spiral localization pattern of FtsA and FtsZ. These PG helical domains may also provide a structure for the attachment of components of the secretory apparatus: SecA, SecY and pre-AmyQ exhibit spiral patterns of localization in *B. subtilis* (Table 1). In the case of SecA, this pattern is dependent on the presence of acidic phosphatidylglycerol phospholipids [44]. On the other hand, the functioning of the *E. coli* SecYEG protein channel complex was shown to be dependent on CL and it is colocalizesed with CL domains [45]. Variability in the structure and lipid composition of membrane domains may therefore be a general factor shaping the interactions of membranes and proteins.

### 3.3. Lipid Rafts

Eukaryotic lipid rafts have been studied for many years. It has been shown that these microdomains are localization targets for some specialized membrane proteins, such as Flotillin-1 or Reggie, which are involved in the regulation of signal transduction, cytoskeleton rearrangement, and vesicle trafficking [48]. Flotillin-reggie proteins are membrane-associated proteins, which carry a SPFH (stomatin, prohibitin, flotillin and HflK/HflC) domain and are present in all types of cells. Flotillin proteins are characterized by the flotillin domain, which is rich in heptad repeats.

Until recently, no lipid raft structures had been characterized in bacteria, though many bacterial membrane proteins, mainly those involved in cell signaling and signal transduction pathways, had been shown to be distributed heterogeneously in the cytoplasmic membrane [49]. Interestingly, most bacterial genomes contain genes for proteins similar to the eukaryotic Flotillin-1 and some of these flotillin-like proteins have been shown to be heterogeneously distributed in the membrane [50,51]. Curiously enough, this punctuate distribution appeared to be independent of the CL and PG domains, implying that another type of lipid domain was present.

Recently it was demonstrated that *B. subtilis* and *Staphylococcus aureus* also form lipid rafts in the membrane and that the lipids associated with these rafts appear to be polyisoprenoids, which are synthesized via pathways involving squalene synthases (Figure 1A) [6]. These authors adapted a method normally used to characterize eukaryotic rafts by partitioning the membranes into detergent-resistant (DRM) and detergent-sensitive (DSM) fractions followed by determining the identities of the proteins in these different fractions. In the DRM fraction, they found sensor kinase KinC, flotilin-like protein FloT, and many other proteins involved in molecule trafficking, cell signaling and protein secretion. Both KinC and FloT were found to colocalize in the membrane at the same foci (Table 1). It was also recently established that bacterial flotillin-like proteins have overlapping functions in a variety of membrane-associated processes and that flotillin domain-mediated assembly, along with NfeD proteins, play an important role in assembling flotillin raft-like structures *in vivo* [46,50,51].

The association of all these different proteins with polyisoprenoid lipids indicates that it should be possible to disrupt the rafts, and therefore the proteins’ functions, using squalene synthase inhibitors; it was, in fact, discovered that the localization of raft-specific proteins and the rafts themselves could be disrupted using the squalene synthase inhibitors zaragozic acid and different kinds of statins [6]. These inhibitors have been shown to block biofilm formation in *B. subtilis* [6] and also inhibit *S. aureus* virulence [52]. This suggests that these lipid rafts can be exploited as new targets for controlling bacterial infection and biofilm formation and that small molecules which inhibit raft formation may be promising antibacterial agents.

## 4. Negative and Positive Curvature Membranes

The manner in which proteins localize on cell membranes is not dependent only on the lipid composition of the membrane or membrane domain. It was recently discovered that bacteria have also evolved completely different mechanisms by which proteins are able to recognize specific sites on the cell membrane, based on the membrane’s different physical rather than chemical characteristics. Two cues have been discovered based on whether the membrane has a negative curvature (concave shape) or positive curvature (convex shape).

The first protein, which was reported to recognize a negative curvature in bacteria was DivIVA. This *B. subtilis* protein is involved in two different functions, one during vegetative growth and a second during sporulation. During cell division, DivIVA recognizes the newly forming cell poles, localizes to and persists at these polar sites, and recruits the other Min system proteins, MinJ, MinD and MinC, which block premature septum formation [31]. DivIVA recruits a different set of proteins to the cell poles during sporulation, when it is required for the proper segregation of sister chromosomes anchored to opposite cell poles by structures close to their *ori*-regions [53–55]. In this sporulation-specific chromosomal arrangement, RacA acts as a bridge between the DivIVA at the cell pole and the *ori* region of the chromosome [56,57]. The apparent switching of binding partners by DivIVA may serve to couple relief of septum formation inhibition to faithful chromosome segregation during sporulation. Interestingly, DivIVA requires no additional proteins, cell division or otherwise, for its polar localization. It was quite a mystery how this protein was able to recognize these specific sites not only in *B. subtilis* but also when expressed heterologously *E. coli* and *Schizosaccharomyces pombe*, both organisms which do not possess a homologue of it [58]. One suggestion was that DivIVA recognized the CL domain at the poles of both *B. subtilis* and *E. coli*, but this failed to explain its polar localization in *S. pombe*. The correct explanation came from the pioneering work of Lenarcic *et al.* [7] and was elaborated further by Ramamurthi and Losick [47]. They found that DivIVA preferably binds to strongly curved cell membranes in cell shape mutants through its *N*-terminus (Figure 1B). In addition, DivIVA is able to distinguish between different degrees of negative membrane curvature and displays a hierarchical preference for the most concave surface available. Thus, it preferentially localizes at septation sites and then to cell poles. This was shown by blocking cytokinesis, causing a redistribution of DivIVA where it is enriched at the poles. In lysozyme treated, spherical cells (protoplasted cells), DivIVA is largely uniformly distributed [47]. DivIVA forms “doggy bone”-like structures of about 22 nm in length, which assemble into large lattices [59]. These doggy-bone structures appear to be composed of 6–8 DivIVA subunits and it was proposed that this relatively large size stabilizes them by “bridging” opposing membranes [7].

Taken together, all these results suggest that a concave surface alone is sufficient for the recruitment of DivIVA to specific sites in the cell. DivIVA homologues from different microorganisms sometimes have different functions and they can recruit different proteins to specific sites. In the non-sporulating Gram-positive cocci *Streptococcus pneumoniae*, which lacks a Min system, a DivIVA homologue is still important for cell division and it interacts with other cell division proteins [60]. In the filamentous actinomycete *Streptomyces coelicolor*, DivIVA is crucial for tip growth and branching [61,62], and interacts with a protein involved in tip growth [63]. In *Corynebacterium glutamicum* and *Mycobacterium tuberculosis*, DivIVA is required for polar growth [64,65]. Interestingly, so far DivIVA is the only protein that has been described in bacterial species, which is able to recognize negative curvature. Thus, it appears that negative curvature is not a widely used cue for membrane protein localization. In the rod shaped *S. pombe*, the Pom1 protein localizes to the cell poles and the unique geometry of this environment suggests that Pom1 may be a eukaryotic example of a protein that recognizes membrane curvature on a cellular scale [66].

In sporulating bacteria such as *B. subtilis*, both convex (positive curvature) and concave membrane shapes can be found (Figure 1C). The formation of convex-shaped membranes is linked to asymmetric cell division, when the cell is divided into a smaller forespore and a larger mother cell. The forespore is subsequently engulfed by the mother cell and its surface is formed by a positively curved membrane, which is recognized by a small, 26 amino-acid long, sporulation specific protein called SpoVM [8]. This protein is likely the first of a large set of coat proteins, which are synthesized in the mother cell and deposited on the forespore surface. SpoVM forms an amphiphatic helix with its hydrophobic face buried in the lipid bilayer [67]. In a mutant in which no positive curvature is formed and SpoVM is still expressed, it localizes to the inner membrane of the mother cell. SpoVM has also been shown to bind to the outer surface of liposomes [8].

## 5. Role of Membrane Potential for Protein Localization

There is an additional physical characteristic of the bacterial cell membrane, which can influence the spatial organization of not only the lipid domains but also the proteins which recognize them. Recently, Strahl and Hamoen showed that the proton motive force (pmf) generated in the membrane can largely delocalize many proteins, which normally recognize the cues described above [9]. They tested more than 20 proteins which show specific localization patterns and which are involved in cell division and its regulation, cell shape regulation, chromosome segregation, signal transduction, secretion and others. A pmf dissipation was achieved by adding the specific proton-ionophore CCCP (carbonyl cyanide *m*-chlorophenylhydrazone). Among the proteins that rapidly changed their localization patterns after incubation with CCCP were those which bind to the membrane transiently through an amphiphatic α-helix, including MinD and FtsA or cytoskeletal proteins that are predicted to be attached to the membrane through other proteins, including MreB, MreBH and Mbl (Table 1). Nevertheless, recently it has also been discovered that both *E. coli* and *T. maritima* MreB interact directly with the cell membrane [68] either through an *N*-terminal amphiphatic helix or via a membrane insertion loop. However, incubation with CCCP had no effect on the localization of proteins recognizing negative curvature, like DivIVA, or those with many transmembrane helices, such as MinJ.

There are two components of pmf, a transmembrane electric potential and a transmebrane proton gradient. It was shown that the electric potential is responsible for this change in protein localization. The change in membrane potential could cause a subtle conformational change in the amphiphatic helix or it may affect the membrane lipid properties, or both. What biological role could the regulation of protein localization through changes in membrane potential have? Bacteria living in different environmental niches, which can change rapidly, could use changes in membrane potential to respond more quickly to environmental challenges. An important environmental factor which can change the membrane potential is the availability of electron acceptors such as oxygen. *B. subtilis* is a soil bacterium and in its natural environment the oxygen level changes constantly. At low oxygen levels, the cells become elongated, possibly as a consequence of the delocalization of the MinD and FtsA proteins [9].

## 6. Changes in Lipid Composition of the Membranes during Sporulation

Some membrane proteins have the ability to change their topology in response to changes in the lipid composition of the membrane, for example, lactose permease LacY from *E. coli* [69]. This protein shows a dual bi-directional reversible topology, which is dependent on the membrane lipid composition. Another documented case of a membrane protein with a dual topology is the L envelope protein of the hepatitis B virus [70]. Many other proteins also show similar characteristics [71,72].

The lipid composition of the membranes can fluctuate greatly during the cell cycle in all organisms from bacteria to humans. A clear example of this can be seen during sporulation in *B. subtilis*. Sporulation is activated by complex regulatory circuits and begins when a threshold concentration of phosphorylated Spo0A is reached. The whole process is closely linked to the cell cycle and it involves an asymmetric cell division. The beginning of sporulation is marked by remarkable changes not only in chromosome partitioning but also in cell division. The sporulation septum bisects the axial filament leaving only one third of one chromosome in the forespore and creating a transient genetic asymmetry [73]. The remaining two-thirds of the chromosome are then transferred from the larger mother cell into the smaller forespore via a conjugation-like mechanism directed by the partitioning protein SpoIIIE [74].

After sporulation begins, Spo0A brings about global changes in gene expression in the cell [75]. During sporulation, gene expression is orchestrated through the activity of four compartment-specific sigma factors (σ^F^, σ^E^, σ^G^ and σ^K^), activated either in the forespore or in the mother cell [76]. These sigma factors, subunits of RNA polymerase, recognize specific sets of promoter sequences and allow transcription of different genes. Recently, it was discovered that Spo0A reactivates *de novo* fatty acid and phospholipids synthesis for membrane biogenesis during transient genetic asymmetry and after asymmetric cell division [14]. These authors suggested that *de novo* lipid synthesis was asymmetrically excluded from the forespore (Figure 1D). This system would allow a different lipid composition to develop on the outer, mother side, of the forespore membrane in contrast to its inner membrane. This was shown to be crucial for the function of SpoIIR, which is produced in the forespore, secreted into the septal membrane, and directionally activates σ^E^ in the mother cell [14]. It is also possible that a difference in lipid composition between the inner and outer membranes of the forespore is important for the function of other membrane proteins. One of these proteins might be SpoIIE, which has a dual function in asymmetric septum formation and in activation of the first compartment specific sigma factor, σ^F^ [77,78]. Other examples of proteins, which might be affected are SpoIIQ and SpoIIIAH, which are important for forespore engulfment.

Even more dramatic rearrangements in the membranes were observed during sporulation of *Acetonoma longum*, an evolutionarily close relative of *Clostrideae* sp. [79]. These authors showed that during sporulation the inner membrane of the mother cell is inverted and transformed to become an outer membrane of the germinating cell. Their results point to sporulation as a mechanism by which the bacterial outer membrane could have arisen. All these factors, taken together, show that dynamic membrane remodeling events during sporulation, which involve differentiated membrane lipid synthesis, may have crucial structural and regulatory roles for the proper localization and function of the proteins involved.

## 7. Conclusion

Many proteins in bacterial cells are compartmentalized and localized in a way, which suggests that these activities are not random, but depend upon certain membrane cues. One of these cues is the presence of certain regions of different lipid composition called membrane domains and they were identified with the help of proteins, which are known to be not dependent on other proteins for their correct localization. However, localization of some of the GFP tagged proteins was recently questioned [80,81] and it will require reevaluation.

Research into the formation of these lipid domains is a relatively new field, but nevertheless it has already given important insights into how proteins recognize specific topological cues and perform their tasks during the cell cycle. Lipid domains, lipid rafts and different physical membrane characteristics have been studied mainly in the two model organisms *E. coli* and *B. subtilis* and these studies have clearly demonstrated that these membrane characteristics are crucial for several important cellular processes, including cell division, signal transduction, sporulation, biofilm formation, chromosome segregation, secretion, and cytoskeleton formation.

In the near future, many interesting revelations about membrane biogenesis in other bacterial species should also come to light. There are still many unanswered questions about the heterogeneity, size, half-lives and dynamics of the membrane cues already characterized. The answers will require the development of new technologies, especially in imaging, as well as the use of multidisciplinary approaches, including elements of biology, chemistry, mathematics and physics.

## Figures and Tables

**Table 1:** Example of proteins recognizing specific cues in the membrane for their localization.

Lipid domain/organization	Proteins	References
Cardiolipin—CL	ProP	[22,23]
Phosphatidylglycerol—PG	MinD, FtsA, SecA	[5,44]
Polyisoprenoids—lipid rafts	FloT, FloA, KinC, YqfA, NfeD2	[6,46]
negative curvature membranes	DivIVA	[7,47]
positive curvature membranes	SpoVM	[8]
rearagement of lipid membrane composition	SpoIIR	[14]
membrane potential	MinD, FtsA, MreB, MreBH, Mbl	[9]
